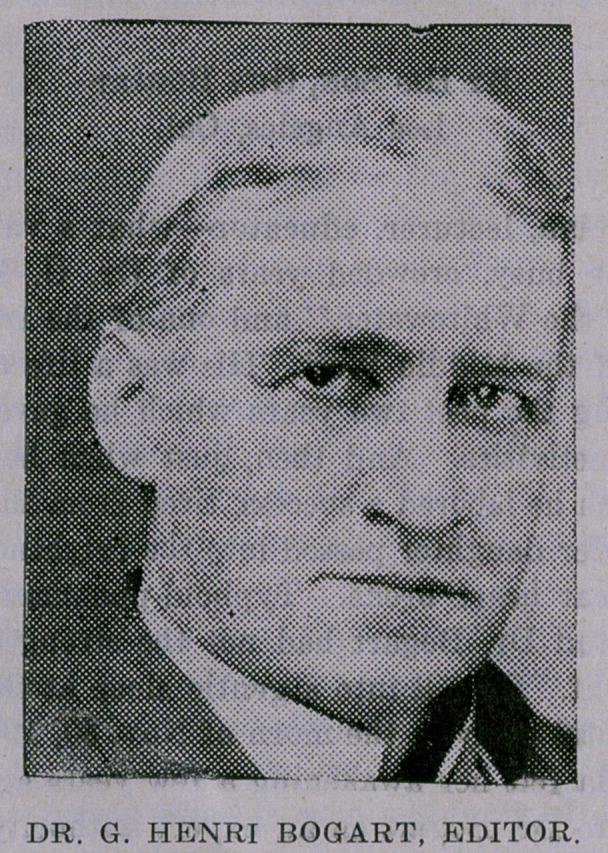# An Appreciation of Dr. G. Henri Bogart

**Published:** 1919-01

**Authors:** Guy Bogart

**Affiliations:** Los Angeles, Cal.


### An Appreciation of Dr. G. Henri Bogart.

By His Son, Guy Bogart
Los Angeles, Cal.

Poet, physician, lecturer, educator—what a varied field was covered in the busy, crowded years of Dr. G. Henri Bogart, "The Poet of the Whitewater," and one of the truest and most typical sons of the Middle West. His was a life of service—intensified during the past two years which he gave unreservedly to war work activities. And then, just when he had seen the cause for which he labored a success, tired from the heavy strain—when his older daughter passed into the brightness of the next world, his great heart broke. Typhoid-pneumonia supervened her funeral, and Dr. Bogart had not the strength nor the will further to fight. He fell into a gentle sleep at the Shelbyville, Illinois, Hospital early in the morn of Nov. 30, 1918.

When Russia felt her awakening a few years ago, the government turned to Dr. Bogart as an expert authority in eugenics: Canada sent to him for advice in drafting Dominion laws; in a dozen states he was consulted as an internationally famous authority in eugenics. And yet, such was his modesty that the facts were never widely proclaimed. As a contributor to a score of the nation's best medical journals—on the staff of several—he was favorably known to the inner circles of the profession.

Born in Ohio, living the greater part of his days in Indiana, and closing his earthly career in Illinois, Dr. Bogart was the embodiment of the heart of the Middle West. Facts and details of this giant's career but feebly convey the story of his life—

for that was lived in the hearts of the obscure and the "common" people.

Dr. G. Henri Bogart was born in Cincinatti, Ohio, October 26, 1857. He attended school at Mt. Airy, graduating from high school at 12 years of age. He then moved to the banks of the Wabash, where he learned a trade, taught school, railroaded, studied medicine and was married—all before he was twenty-one. A large part of his life was spent in the pioneer town of Brookville, Ind. Of this period Alonza Rice wrote in a little volume, "Some Indiana Writers and Poets":

"Old Brookville appeals to something that makes men dream dreams, and he has a quaint, wide home in the edge of that historic town; a home hidden in roses and woodbine, with the laugh-

DR. G. HENRI BOGART, EDITOR.

ing, leaping river singing thru its garden, and the free wild song birds winging thru its greenery."

Sacrificing his own desires, Dr. Bogart spent many years in giving his children an education. After the Brookville years came a period of newspaper work in Terre Haute, Indiana and in Champaign, Paris and Shelbyville, Illinois.

Dr. G. Henri Bogart was one of the most versatile and unique figures in the magazine field. He came from a race of teachers and doctors, his French-Holland ancestor having come to New Amsterdam in 1616 as the surgeon of the Dutch West India company. The same record is true on the maternal side. Dr. Bogart was a graduate of both the allopath and eclectic schools of medicine—but, as in everything, he was an original thinker and not a follower of doctrines. He early became a free lance in life—with his pen ever busied for the advancement of universal freedom.

He was from boyhood an omnivorous reader and possessed a photographic memory that constantly surprised his friends.

It is as a poet that Dr. Bogart achieved his best. His only published book was a slender volume, "Some Songs by an Optimist." He was one of the early members of the Western Association of Writers and actively associated with James Whitcomb Riley, Strickland Gillilan, Benj. S. Barker and such lights of the literary world. When the Indiana State Board of Education desired representative Hoosier poets presented to the school children, he was included in the thirty-seven selected to represent the myriad writers of the State. He was included in numerous anthologics of middle western writers.

The doctor was a prolific writer and his "Song Sermons" and other contributions were widely syndicated. There is a wondrous lyrical quality and a depth of sentiment that covers the whole range of human emotions in his poems.

All that is mortal of this beloved lover, this champion of progress and truth rests beside his devoted daughter in Lebanon, Indiana. His untrammeled soul is still "carrying on" the work that undermined his health.

Other men were more popularly known, but there is not a state in the Union, but will number its quota of friends who will mourn for the big-hearted, brave, chivalrous friend of humanity.

## Figures and Tables

**Figure f1:**